# Overexpression of YEATS2 Remodels the Extracellular Matrix to Promote Hepatocellular Carcinoma Progression via the PI3K/AKT Pathway

**DOI:** 10.3390/cancers15061850

**Published:** 2023-03-20

**Authors:** Xin Liu, Yi Hu, Cairong Li, Jiayu Chen, Xiaohong Liu, Yang Shen, Yangtao Xu, Wenliang Chen, Ximing Xu

**Affiliations:** 1 Department of Oncology, Renmin Hospital of Wuhan University, Wuhan 430060, China; 2 Department of Oncology, General Hospital of Central Theater Command, Wuhan 430061, China; 3 School of Clinical Medicine, Xianning Medical College, Hubei University of Science and Technology, Xianning 437100, China

**Keywords:** YEATS2, hepatocellular carcinoma, proliferation, metastasis, PI3K/AKT pathway, extracellular matrix

## Abstract

**Simple Summary:**

Hepatocellular carcinoma (HCC) is a malignant tumor with a high incidence rate and the fourth leading cause of death in cancer patients. YEATS domain containing 2 (YEATS2) gene encodes a scaffolding subunit of the ATAC complex. We found that YEATS2 was upregulated in HCC tissues and was associated with poorer prognosis of patients. We found that overexpression of YEATS2 promoted the process of tumor proliferation, migration, and invasion. Mechanistically, we revealed that YEATS2 promoted liver cancer progression by activating the PI3K/AKT signaling pathway and remodeling the extracellular matrix. Therefore, these findings suggest that YEATS2 holds promise as a new therapeutic target.

**Abstract:**

Hepatocellular carcinoma (HCC) is one of the most common cancers and the fourth leading cause of death in men. YEATS domain containing 2 (YEATS2) gene encodes a scaffolding subunit of the ATAC complex. We found that YEATS2 was upregulated in HCC tissues and was associated with a poor prognosis. However, the role of YEATS2 in HCC remains unclear. The purpose of this study was to investigate the effect of YEATS2 on the progression of HCC and to elucidate its related mechanisms. We found that overexpression of YEATS2 promoted tumor cell proliferation, migration, and invasion through the PI3K/AKT signaling pathway and regulation of extracellular matrix. These findings help to understand the role of YEATS2 in HCC, and YEATS2 may become a new target for HCC therapy.

## 1. Introduction

Hepatocellular carcinoma is the fourth leading cause of cancer-related death in the world, with an estimated incidence of over 1 million by 2025 [1,2]. Compared with traditional chemotherapy, targeted therapy has a better survival rate and fewer side effects in patients with intermediate–advanced liver cancer [3]. However, due to the high heterogeneity of the tumor, the drug will gradually lose its effect. Therefore, it is crucial to explore the underlying mechanisms of HCC progression and identify new molecular targets.

YEATS domain containing 2 (YEATS2) protein is a scaffolding subunit of the ATAC complex [4,5,6]. Studies have shown that YEATS2 is a selective histone crotonylation reader and is related to histone acetylation [7,8]. In addition, YEATS2 has been shown to be associated with the progression of various tumors. Overexpression of YEATS2 promotes the proliferation and migration of pancreatic cancer cells [9,10]. YEATS2 has been reported to be associated with the progression of non-small-cell lung cancer through histone acetylation [8]. In addition, knockout of the YEATS2 gene reduced the migration and invasion ability of HNSCC cells [11]. Overall, YEATS2 is closely related to cancer progression, but the role of YEATS2 in liver cancer is still unclear.

In the study, we found that YEATS2 is upregulated in liver cancer. In addition, YEATS2 overexpression significantly promoted the proliferation, migration, and metastasis of HCC cells. In addition, YEATS2 overexpression activates the PI3K/AKT pathway and remodels the extracellular matrix, promoting the proliferation, migration, and metastasis of HCC cells. Taken together, our findings suggest that the upregulation of YEATS2 is critical for the progression of HCC, which provides new ideas for future treatment of HCC.

## 2. Materials and Methods

### 2.1. Bioinformatics Analysis

We used data from our team’s previous microarray analysis of 5 paired tissue samples [12]. We downloaded sample expression profiles and corresponding clinical information data from TCGA database (https://portal.gdc.cancer.gov/, accessed on 10 May 2022) and GEO database (https://www.ncbi.nlm.nih.gov/geo/, accessed on 11 May 2022). Protein-coding genes were annotated using the GENCODE project.

By using the “limma” package in R software (version 4.1.3), we analyzed the expression of YEATS2 in liver cancer samples and normal liver tissue samples and the correlation between YEATS2 expression with clinical characteristics. Graphs were drawn with the “ggpubr” package. Survival curves were constructed using the Kaplan–Meier method, with group cutoffs set to the median of YEATS2 expression.

We utilized the “limma” package to analyze differentially expressed genes in the high-expression YEATS2 group and low-expression YEATS2 group. False discovery rate (FDR) values < 0.05 and |Log FC > 1| were used as cutoffs. The “clusterProfiler” package in R software was used for GO analysis and KEGG pathway enrichment analysis of differentially expressed genes. The AmiGO2 online data website (http://amigo.geneontology.org/, accessed on 24 November 2022) was used to query specific genes under enriched GO category.

### 2.2. Cell Culture

HCC cell lines (MHCC97H and SMMC-7721) were purchased from Procell Life Science & Technology Co., Ltd. (Wuhan, China). The cell lines were cultured in Dulbecco’s modified Eagle’s medium (DMEM) supplemented with 10% fetal bovine serum (FBS; Thermo Fisher Scientific, Inc., Waltham, MA, USA). All cells were cultured in a humidified incubator at 37 °C in the presence of 5% CO_2_.

### 2.3. Western Blotting

Cell samples were lysed with protease inhibitors and phosphatase inhibitors in RIPA lysis buffer on ice. After centrifugation at 12,000 rpm for 20 min, the supernatant of each sample was collected and the concentration of protein in the sample was detected using the BCA assay. All protein samples were stored at −80 or −20 °C. Proteins in each sample were separated using a 10% SDS polyacrylamide gel at 80 V and transferred to PVDF membranes (Millipore, NJ, USA) at a current of 200 mA. The membranes were blocked by 5% skim milk for 1 h at room temperature and incubated with the corresponding primary antibody overnight at 4 °C. Anti-rabbit or anti-mouse horseradish peroxidase (HRP)-labeled secondary antibodies were incubated at room temperature for 50 min, and the bands were visualized with a chemiluminescent kit. The primary antibodies are listed in Table 1. The uncropped blots and molecular weight markers are shown in Supplementary Figure S1.

### 2.4. Cell Transfection Assay

To construct the YEATS2 overexpression model, a YEATS2 overexpression plasmid (pCMV-hYEATS2) (Miaoling Biotechnology, Wuhan, China) was constructed. Transient transfection was performed using Lipofectamine 8000 (Beyotime Biotechnology, Shanghai, China) when the cell density was 60–70%. Transfection efficiency was determined by Western blotting analysis.

### 2.5. Cell Proliferation Assay

Liver cancer cells were seeded in 96-well plates at a density of 3000 cells per well. A medium containing 10 uL CCK-8 (Servicebio, Wuhan, China) was added to each well. After incubation for 2 h at 37 °C, the absorbance of each well was measured at 450 nm. In addition, cell proliferation was detected using Ki67 immunofluorescence.

### 2.6. Wound Healing Assay

Cells were seeded in 6-well plates and scraped vertically with a 200 μL pipette when the cells reached approximately 90% confluency. After washing three times with PBS, culture was continued with a medium containing 2% FBS. Images were collected using a microscope at 0 and 24 h after scratching.

### 2.7. Transwell Migration and Invasion Assays

Serum-free DMEM cells (5 × 10^4^ cells/well), pre-starved for 12 h, were seeded in the upper chamber of the Transwell plate, and DMEM containing 20% FBS was added to the lower chamber. After 24 h of incubation, the remaining cells in the upper chamber were removed with a cotton swab, and the invading cells were fixed with formalin and stained with crystal violet for 15 min. Cells were counted under an inverted microscope (Olympus Corporation) at 200× magnification. For the invasion assay, 70 μL Matrigel (BD Biosciences, San Jose, CA, USA) was used to pre-coat Transwell chambers at 37 °C for 4 h, and the above steps were repeated.

### 2.8. Immunofluorescence

HCC cells were seeded on sterile glass slides, and after reaching 75% confluency, the medium was removed, and then the cells were washed with PBS and fixed with paraformaldehyde for 20 min. They were blocked with goat serum and incubated with the primary antibody overnight at 4 °C. Cells were incubated with fluorescently labeled secondary antibodies for an additional 60 min, and finally, nuclei were stained with DAPI.

### 2.9. Flow Cytometry

For cell cycle analysis, cells washed three times with pre-cooled PBS were collected in centrifuge tubes, fixed with 70% ethanol, and incubated overnight at 4 °C. After washing with PBS again, PI was added, and then the suspension cells were subjected to cell cycle detection.

### 2.10. Statistical Analysis

Statistical analysis was performed using GraphPad Prism 9. YEATS2 expression data are presented as median and interquartile ranges in bioinformatics analysis. Data from experiments are presented as mean ± SD. The data conforming to the normal distribution and homogeneity of variance were used for *t*-test or ANOVA test. Ranked data and the data not obeying the normal distribution and homogeneity of variance were used by non-parametric test. Statistical significance was defined as *p* < 0.05.

## 3. Results

### 3.1. Expression and Prognosis Analysis of YEATS2 in Liver Cancer

**Figure 1:**
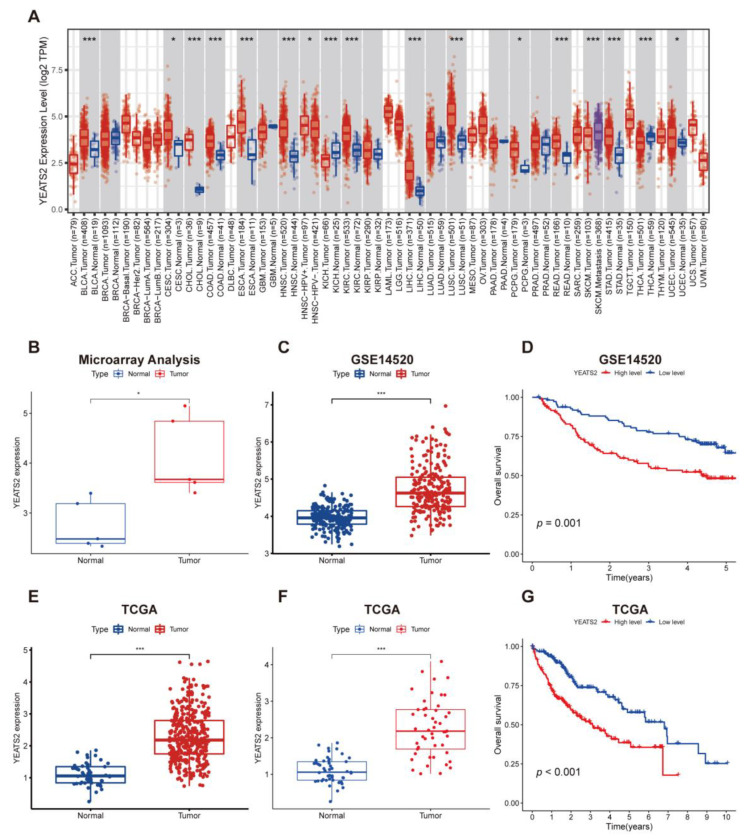
Expression characteristics of YEATS2 in liver cancer samples and its relationship with clinical prognosis. (**A**) Expression of YEATS2 in multiple tumors in the TIMER database. (**B**) Expression of YEATS2 in microarray data analysis of paired samples. (**C**) In the GSE14520 dataset, the expression level of YEATS2 was higher in HCC tissues compared with normal liver tissues. (**D**) Kaplan–Meier analysis was performed on the overall survival (OS) of HCC patients according to the expression of YEATS2 in the GSE14520 dataset. (**E**) YEATS2 expression was identified in 373 tumors and 49 normal samples from TCGA. (**F**) Expression of YEATS2 in paired samples from TCGA. (**G**) Kaplan–Meier analysis was performed on the overall survival (OS) of HCC patients according to the expression of YEATS2 in the TCGA dataset. Data are presented as median and interquartile ranges. * *p* < 0.05, *** *p* < 0.001.

To understand the role of YEATS2 in tumors, we first analyzed its expression level in tumors and healthy tissue pairs of several different malignancies. The Timer database showed that the expression level of YEATS2 was upregulated in various tumors compared with normal samples (Figure 1A). Furthermore, in our team’s previous microarray data analysis of tissue samples, it was shown that the expression of YEATS2 in liver cancer was higher than that in normal samples (Figure 1B). According to the GSE14520 dataset, the expression of YEATS2 in liver cancer tissues was significantly higher than that in normal tissues, and patients with high expression of YEATS2 had worse prognoses (Figure 1C,D). In addition, the TCGA database showed that the expression of YEATS2 in 373 liver cancer tissues was higher than normal samples (Figure 1E). Analysis of paired samples in TCGA database also showed that YEATS2 was upregulated in HCC tissues compared with adjacent tumor tissues (Figure 1F). TCGA data also showed that patients with high expression of YEATS2 had worse prognoses (Figure 1G).

### 3.2. Correlation between YEATS2 Expression and Clinical Features in Liver Cancer

**Figure 2:**
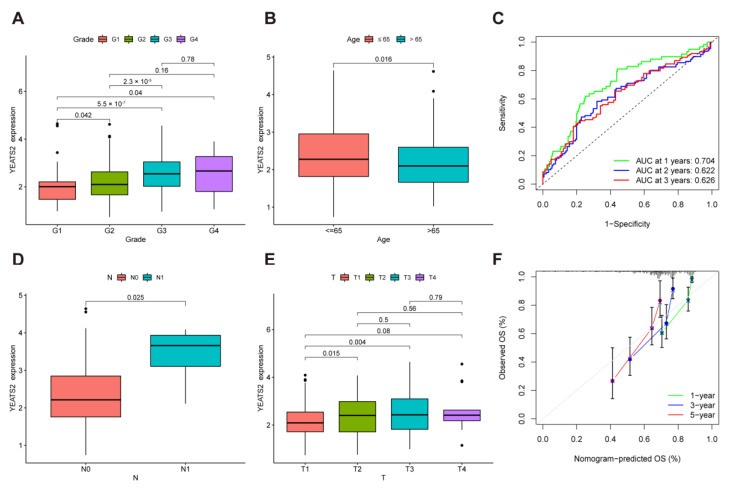
Correlation analysis between YEATS2 expression and clinical features of liver cancer. (**A**) Relationship between YEATS2 expression and grades. (**B**) Relationship between YEATS2 expression and ages. (**C**) Time-dependent ROC analysis of YEATS2. (**D**) Relationship between YEATS2 expression and lymph node metastasis. (**E**) Relationship between YEATS2 expression and T stages. (**F**) Nomogram analysis of YEATS2. Data are presented as median and interquartile ranges.

We further explored the relationship between YEATS2 and clinical features. We found there was significant difference between grade 1 and grade 2 (*p* = 0.042), grade 1 and grade 3 (*p* < 0.001), grade 1 and grade 4 (*p* = 0.04), and grade 2 and grade 3 (*p* < 0.001) (Figure 2A). Patients aged ≤ 65 had higher YEATS2 expression (*p* = 0.016) (Figure 2B). According to the result of the time-dependent receiver-operating characteristic curve (time-dependent ROC) analysis, the area under the curve (AUC) of YEATS2 at 1, 2, and 3 years was 0.704, 0.622, and 0.626, indicating that YEATS2 has better predictive survival ability in liver cancer (Figure 2C). Further, we analyzed the relationship between YEATS2 expression and lymph node metastasis. The results showed that patients with N1 lymph node metastasis had higher YEATS2 expression than N0 patients without lymph node metastasis (Figure 2D). There was a significant difference between T1 and T2 (*p* = 0.015) as well as T1 and T3 (*p* = 0.004) (Figure 2E). In addition, the nomogram shows that YEATS2 has good consistency in predicting OS (Figure 2F).

### 3.3. Overexpression of YEATS2 Promotes HCC Cell Proliferation

**Figure 3:**
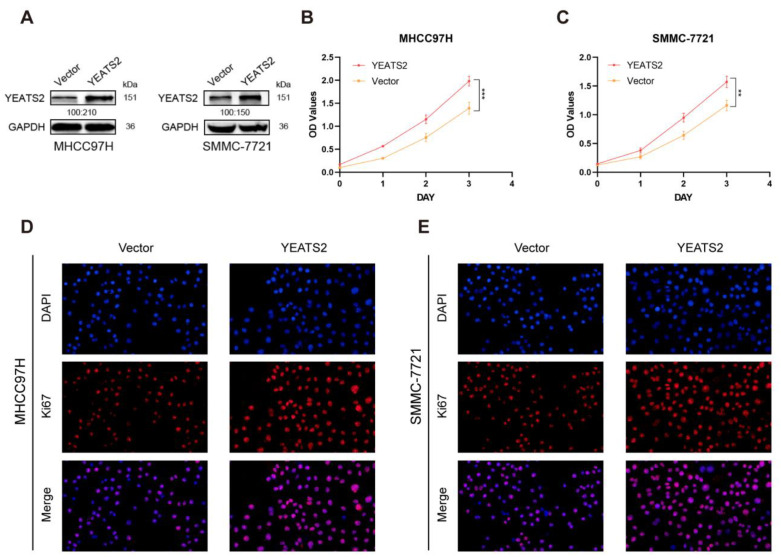
YEATS2 overexpression promoted the proliferation of liver cancer cells. (**A**) Western blot experiments confirmed the effect of YEATS2 overexpression. (**B**,**C**) The CCK8 assay showed that YEATS2 overexpression promoted cell proliferation. (**D**,**E**) Detection of Ki67 expression in cells using immunofluorescence. ** *p* < 0.01, *** *p* < 0.001.

Subsequently, we further explored the effect of YEATS2 on the biological behavior of liver cancer cells MHCC97H and SMMC-7721. The results of Western blotting showed that the expression of YEATS2 in the pc-YEATS2 group was higher than that in the pc-vector group (Figure 3A). The CCK-8 experiment showed that compared with the pc-Vector group, the proliferation ability of the cells in the pc-YEATS2 group was significantly enhanced, indicating that the overexpression of YEATS2 enhanced the growth of liver cancer cells (Figure 3B,C). In addition, the results of Ki67 immunofluorescence test also showed that the expression of Ki67 in the cells in the high-expression YEATS2 group was higher than in the cells in the pc-Vector group (Figure 3D,E). This further confirmed that YEATS2 had a pro-proliferative effect on liver cancer cells.

### 3.4. Effects of YEATS2 on Migration, Invasion, and Cycle of Hepatocellular Carcinoma Cells

**Figure 4:**
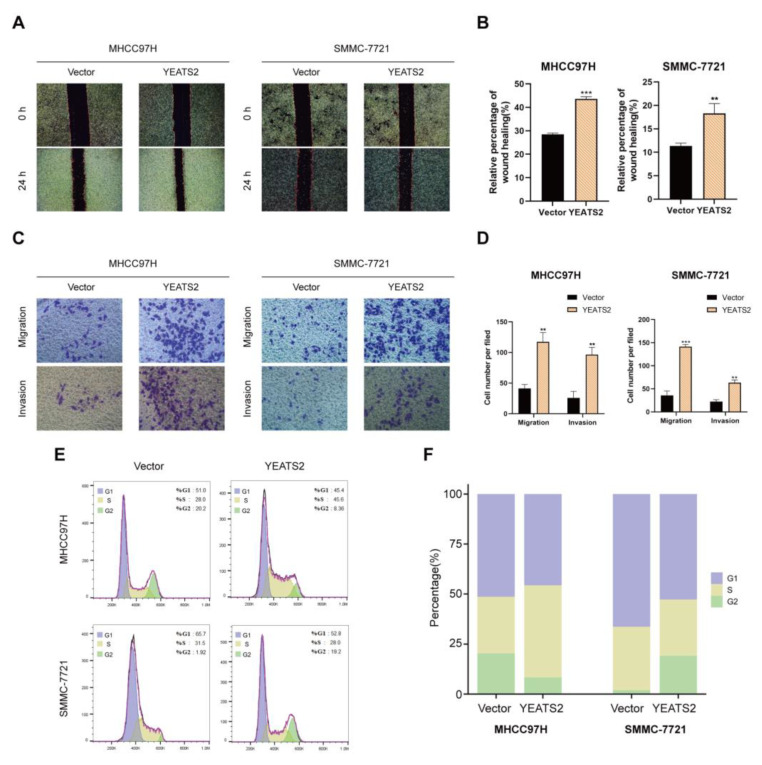
YEATS2 overexpression promotes migration, invasion, and cycle of liver cancer cells. (**A**,**B**) Cell migration ability was analyzed using the woundhealing assay. (**C**,**D**) Assessing cell migration and invasion ability using Transwell assays. (**E**,**F**) The effect of YEATS2 on the cell cycle of MHCC97H and SMMC-7721 was detected by flow cytometry. Data are expressed as the mean ± SD. ** *p* < 0.01, *** *p* < 0.001.

Wound healing assays showed that a high expression of YEATS2 enhanced the migration ability of MHCC97H and SMMC-7721 cells (Figure 4A,B). Further, Transwell migration and invasion assays showed that YEATS2 overexpression increased the number of cells that invaded the chambers (Figure 4C,D). These findings suggested that YEATS2 overexpression promoted the migration and invasion of HCC cells. In addition, we detected the cell cycle changes in MHCC97H and SMMC-7721 cells after YEATS2 overexpression using flow cytometry. The results showed that in the PC-YEATS2 group, the proportion of cells in the G1 phase was significantly reduced in MHCC97H and SMMC-7721 cell lines (Figure 4E,F). Collectively, the findings showed that YEATS2 overexpression promoted the migration, invasion, and cycle of liver cancer cells.

### 3.5. YEATS2 Activates PI3K/AKT Pathway and Mediates MMP7 to Remodel Extracellular Matrix

**Figure 5:**
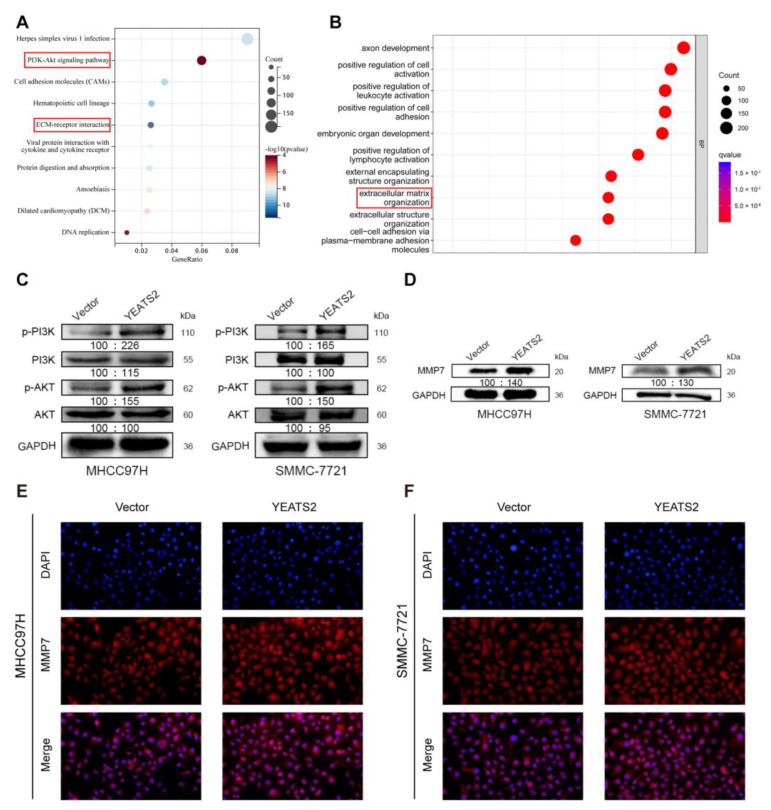
YEATS2 activates PI3K/AKT signaling pathway and remodels extracellular matrix. (**A**,**B**) GO and KEGG pathway enrichment analysis results indicated that YEATS2 participates in the progression of liver cancer through PI3K/AKT pathway and regulating extracellular matrix. (**C**) Western blot analysis of p-PI3K and p-AKT expression. (**D**) Western blot analysis of MMP7 expression. (**E**,**F**) Detection of MMP7 expression in cells using immunofluorescence.

To explore how YEATS2 participates in the progression of liver cancer, we first performed GO analysis and KEGG pathway enrichment analysis on the differentially expressed genes in the high-expression YEATS2 group and low-expression YEATS2 group. Enrichment analysis results showed that YEATS2 may participate in the progression of liver cancer through the PI3K/AKT pathway and extracellular matrix organization (Figure 5A,B). Western blotting was used to validate the above enrichment analysis results. Phosphorylated PI3K and phosphorylated Akt decreased, while no significant changes were observed in T-PI3K or T-Akt protein levels (Figure 5C). After querying the AmiGO2 online data website (http://amigo.geneontology.org/, accessed on 24 November 2022), we found that MMP7 was among the genes from the “extracellular matrix organization” category. In addition, MMP7 is a member of the matrix metalloproteinases (MMPs) family involved in the degradation and remodeling of the extracellular matrix [13,14]. The results showed that the protein expression levels of MMP7 were increased in the YEATS2 overexpression groups (Figure 5D). Moreover, the results of MMP7 immunofluorescence detection also showed that the expression of MMP7 in the cells of the high-expression YEATS2 group was higher than that of the cells of the pc-Vector group (Figure 5E,F).

### 3.6. YEATS2 Regulates the Expression of MMP7 through the PI3K/Akt Pathway to Remodel the Extracellular Matrix

**Figure 6:**
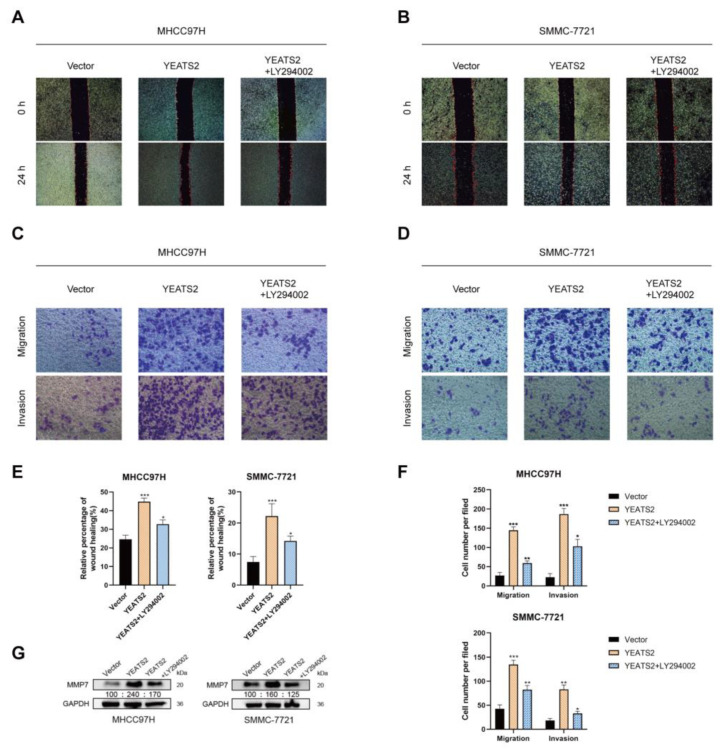
YEATS2 affects the migration and invasion of liver cancer cells through the PI3K/AKT/MMP7 pathway. (**A**,**B**,**E**) The PI3K inhibitor LY294002 partially reversed the effect of YEATS2 overexpression on cell migration in wound healing assays. (**C**,**D**,**F**) In the Transwell experiments, the PI3K inhibitor LY294002 partially restored the effect of YEATS2 overexpression on cell migration and invasion. (**G**) Western blot analysis of MMP7 expression after using LY294002. Data are expressed as the mean ± SD. * *p* < 0.05, ** *p* < 0.01, *** *p* < 0.001.

**Figure 7:**
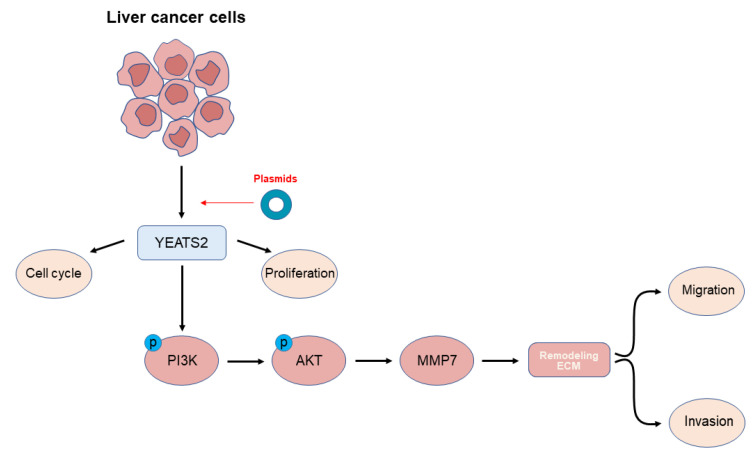
Overexpression of YEATS2 remodels ECM to promote Hepatocellular carcinoma progression via the PI3K/AKT/MMP7 pathway.

MMPs have been reported to promote tumor progression and metastasis by degrading the extracellular matrix and enabling tumor cell migration and invasion [15,16]. We also learned that the expression of MMP7 may be related to the PI3K/Akt pathway [17,18]. In order to observe the effect of the PI3K/AKT pathway on the expression of MMP7 and the ability of migration and invasion of hepatoma cells, MHCC97H and SMMC-7721 cells were treated with PI3K/AKT inhibitor LY294002 (20 μM) for 24 h. In wound healing experiments, LY294002 partially counteracted the promoting effect of YEATS2 overexpression on the migration of liver cancer cells (Figure 6A,B,E). In Transwell assays, LY294002 was able to reverse the promoting effect of YEATS2 on cell migration and invasion (Figure 6C,D,F). We further explored the changes in MMP7 protein expression after using LY294002. LY294002 can partially antagonize the change in MMP7 expression (Figure 6G). Based on the above results, YEATS2 can promote the progression of HCC by regulating MMP7 through the PI3K/AKT signaling pathway (Figure 7).

## 4. Discussion

According to statistics, liver cancer is the fifth most common fatal malignant tumor in the United States [19,20]. Although the treatment methods for liver cancer patients are increasing, the prognosis of liver cancer patients is still poor [21,22]. Tumor heterogeneity and drug resistance are common causes of cancer treatment failure, which account for the majority of cancer-related deaths [23,24]. Therefore, it is particularly urgent and important to screen new liver cancer biomarkers. In this study, we first found that YEATS2 was upregulated in HCC and promoted HCC progression through the PI3K/Akt/pathway. Our findings reveal a novel mechanism by which YEATS2 regulates tumor progression.

YEATS2 protein is a scaffolding subunit of the ATAC complex. However, the current literature on the role of YEATS2 is sparse, covering only a few types of tumors. It has been reported that YEATS2 can act as a target of HIF1α and regulate the TAK1/NF-κB pathway to promote tumor progression in pancreatic cancer [9,10]. In addition, studies have shown that YEATS2 is related to the occurrence of non-small-cell lung cancer [8]. A study finds YEATS2 may be linked to poor prognoses in Wilms tumors [25]. It has been reported that YEATS2 may function as a methylation driver gene in cancer patients with smoking history [26]. However, the role of YEATS2 in HCC remains unclear. Through GEO and TCGA database analysis, we found that YEATS2 was significantly highly expressed in liver cancer, which was consistent with our previous microarray analysis data of liver cancer tissue samples [12]. We also found that YEATS2 expression was associated with poorer prognosis in HCC. How YEATS2 specifically functions in liver cancer is the next goal of this study.

Our study showed that YEATS2 expression promoted the proliferation, migration, and invasion of HCC cell lines MHCC97H and SMMC-7721. YEATS2 also reduced the proportion of the G1 phase of the cell cycle, indicating active cell division. Further, we explored the mechanism of YEATS2 on HCC progression using GO and KEGG pathway enrichment analysis. The results suggested that YEATS2 was related to the PI3K/AKT signaling pathway and extracellular matrix (ECM). Activation of the PI3K/AKT pathway is associated with the malignancy of a variety of tumors, including gastric cancer, leukemia, prostate cancer, and breast cancer [27,28,29,30]. PI3K/AKT and downstream molecule mTOR can promote tumor proliferation, survival, metastasis, and invasion, and inhibit autophagy and senescence [31,32]. The extracellular matrix is a highly dynamic structure whose dysregulation leads to tumor progression. ECM can regulate cell growth, migration and differentiation, vascular development, and immune function. The dynamic process of ECM is mediated by matrix-degrading enzymes, including matrix metalloproteinases (MMPs) [33,34]. MMPs could degrade extracellular matrix components, release cytokines, and recruit immune cells to cause uncontrolled tumor growth, local invasion, and metastasis [13,35]. Taken together, these findings suggest that the PI3K/AKT pathway, ECM, and MMPs are key links in tumor progression.

Our study showed that overexpression of YEATS2 significantly increased the expression of p-PI3K, p-Akt, suggesting that YEATS2 can regulate tumor progression through the PI3K/AKT signaling pathway. In addition, Western blot analysis also showed that YEATS2 can regulate the expression of MMP7, an important member of MMPs. Our subsequent recovery experiments found that the PI3K inhibitor LY294002 reversed the promotion effect of YEATS2 on the migration and invasion of liver cancer cells and the expression of MMP7.

Although it has been confirmed in many studies that the PI3K/AKT pathway can regulate the expression of the MMP family, some studies have shown that the PI3K inhibitor LY294002 can not only inhibit the expression of PI3K but may also act on other proteins [18,36]. This indicates that LY294002 may also regulate the changes in the MMP family through other pathways, which need to be determined in future studies.

In summary, YEATS2 can affect tumor progression by regulating MMP7 expression through the PI3K/AKT signaling pathway. Therefore, our study suggests that YEATS2 may serve as a new therapeutic target in the future.

## 5. Conclusions

In conclusion, YEATS2 was overexpressed in HCC patients and was significantly associated with poor prognosis in HCC patients. YEATS2 acts through the PI3K/AKT/MMP7 signaling pathway and affects the progression of liver cancer. Targeting YEATS2 has the potential to be used as a treatment for liver cancer. However, further studies are needed to provide insight into the mechanism and to expand its clinical application.

## Figures and Tables

**Table 1:** All the primary antibodies used in the study.

Antibody	Specificity	WB	IF	Company
YEATS2	Rabbit	1:1000	-	Proteintech
PI3K	Rabbit	1:1000	-	Affinity
p-PI3K	Rabbit	1:1000	-	Bioss
AKT	Rabbit	1:1000	-	ABclonal
p-AKT	Rabbit	1:1000	-	ABclonal
Ki67	Rabbit	-	1:200	Abcam
MMP7	Rabbit	1:1000	1:200	Abcam
GAPDH	Mouse	1:1000	-	Proteintech

## Data Availability

The data presented in this study are available in Supplementary Materials here.

## Supplementary Materials

The following supporting information can be downloaded at: https://www.mdpi.com/article/10.3390/cancers15061850/s1, Figure S1: Whole Western blot in the study.
